# Black-focused social prescribing:the importance of an Afrocentric approach

**DOI:** 10.24095/hpcdp.44.6.07

**Published:** 2024-06

**Authors:** Sofia Ramirez, Natasha Beaudin, Jennifer Rayner, Neil Price, Daniel Townsend

**Affiliations:** 1 Alliance for Healthier Communities, Toronto, Ontario, Canada; 2 Centre for Studies in Family Medicine, Faculty of Health Sciences, Western University, London, Ontario, Canada; 3 LogicalOutcomes, Toronto, Ontario, Canada

**Keywords:** Black-focused social prescribing, Afrocentricity, holistic health, anti-Black racism

## Abstract

The Black-Focused Social Prescribing (BFSP) project is a unique initiative by the Alliance for Healthier Communities that intertwines Afrocentric principles with social prescribing. Going beyond conventional social prescribing models, BFSP addresses specific health needs within Black communities. It is rooted in the Alliance Black Health Strategy, advocates for Black health, and is guided by Afrocentric principles. The evaluation framework prioritizes client voices, ensuring cultural safety and, by taking time for trust-building, underscores the importance of an inclusive approach. BFSP holds the potential to foster community trust and engagement, and enhance health outcomes in the Black community.

**Highlights**

- Social prescribing is a health care approach that connects social and clinical aspects of health.
- Ensuring access to services without discrimination is crucial for improving the health of Black people in Ontario.
- Tailored interventions are increasingly recognized as necessary to address challenges faced by diverse ethnic and cultural groups.
- Black-focused social prescribing, particularly the Afrocentric approach, aims to enhance the health outcomes of Black individuals.
- Evaluating a Black-focused social prescribing program requires time to create a framework and to consider its nuanced aspects.

## Introduction

Social prescribing integrates social and clinical aspects of health, and recognizes the interconnectedness of physical, mental and social well-being. Through social prescribing, a formal referral pathway documented in the client’s electronic medical record links them to local, nonclinical services to address issues such as social connectedness, mild depression or anxiety. Social prescribing emphasizes a strengths-based approach to co-creating solutions with clients, as well as regular collection of client self-reported experience measures as meaningful data. While social prescribing is effective in various communities, there is a growing acknowledgement of the necessity for tailored interventions addressing the unique experiences of ethnic and cultural populations.^1^ In this article, we delve into the importance of an Afrocentric approach within Black-focused social prescribing (BFSP), emphasizing cultural context and community focus in improving the health outcomes of Black individuals.

## Rx: Community—Social Prescribing pilot project

The Alliance for Healthier Communities, a network of community-governed, team-based primary health care organizations in Ontario, conducted Rx: Community—Social Prescribing,^2^ Canada’s first social prescribing research project, from 2018 to 2020. Over 1100 adult clients participated in the pilot at 11 community health centres. Nearly half were aged 61 to 80 years old and over a third identified as non-White. A mixed-methods evaluation was implemented using pre- and post-intervention surveys and focus groups to assess key themes and changes in self-reported health outcomes. Participants reported reduced stress and anxiety, increased self-confidence and purpose, and improved health management skills. The pilot demonstrated improvements in client well-being, including perceived decreases in loneliness and mental health, and an increase in participation in social activities. Repeat visits to clinicians also decreased.^2^

## The Black Health Committee and Black Health Strategy

In 2018, Ontario’s Black health leaders established the Black Health Committee to leverage their roles in community health organizations for enhancing health outcomes among Black communities in Ontario. The Black Health Committee created the Black Health Strategy, an evidence-informed policy framework integral to the vision of advancing Black health. It outlines foundational processes to ensure equitable health standards for Black individuals accessing care in Ontario.

The Black Health Strategy’s work informed the BFSP project, recognizing that social prescribing could be a valuable tool to address the unique needs of the Black population by incorporating cultural competence and targeted interventions that consider the historical and systemic factors influencing health and well-being in the Black community.^3^

## Black-focused social prescribing project

Building on the learnings from the pilot and using the Black Health Strategy, the Alliance created the BFSP project. Community health centres (CHCs) with a proven record of supporting Black clients were selected to develop a social prescribing model for clients of all ages grounded in Afrocentric values and principles to provide a framework to spread this work. These CHCs are Black Creek, Rexdale and TAIBU in Toronto, and Somerset West in Ottawa.

***Goals of the project:***

The Black-Focused Social Prescribing project charter lists the three main goals of the project:^4^

(1) to develop a BFSP model based on culturally safe values and principles with data collection and evaluation, in order to understand the processes and impacts;

(2) to foster multisectoral conversations and innovative partnerships on culturally specific social prescribing; and 

(3) to widen and deepen learning networks and public awareness. This project offers unique insights and deepens the conversation on considerations for cultural safety within social prescribing.

## The Afrocentric perspective

Afrocentricity emphasizes the importance of cultural context and recognizes the values and historical experiences of people of African descent. It acknowledges that these experiences shape Black communities’ unique health needs and concerns.

An Afrocentric approach aims to celebrate and reinforce cultural identity within the Black communities by connecting individuals with culturally relevant resources, activities and support networks. An Afrocentric approach can be used to provide holistic, culturally appropriate health care.^5^

## Traditional knowledge

When creating programming for BFSP in a local community served by a CHC, taking into account the values of the different communities ensures that participants feel and share a sense of belonging. To ensure that programming reflects the local community’s cultural values, staff from the CHCs held a peer-led session to learn about Afrocentric perspectives, including the seven principles of Kwanzaa, which are derived from the Swahili language:^6^

(1) *Umoja* (unity): to strive for unity within family, community, nation and race;

(2) *Kujichagulia* (self-determination): to define, name, create and speak for ourselves;

(3) *Ujima* (collective work and responsibility): to build our community together, take on its problems as our own and solve them collectively;

(4) *Ujamaa* (cooperative economics): to establish and maintain joint businesses, stores and shops for shared prosperity;

(5) *Nia* (purpose): to make our collective mission the development and restoration of our community’s traditional greatness;

(6) *Kuumba* (creativity): to constantly enhance our community, leaving it more beautiful and beneficial for future generations; and

(7) *Imani* (faith): to wholeheartedly believe in our people.

Four guiding principles were selected by the BFSP Steering Committee, through peer discussion, to guide the work; therefore unity, purpose, self-determination and creativity are infused in all BFSP work.

## Culture as a social prescription

Culture as a social prescription aims to connect people to the vibrancy and strengths of their culture and, in doing so, enhance their overall health.

The following are examples of cultural prescriptions:

- Forty-five clients attended the play *Da Kink in My Hair* to foster social connection. The event, including a meal at a Black-owned restaurant, prompted discussions on content and coping strategies. Post-event feedback highlighted the value of Black representation and emphasized the need for more shared Black stories to contribute to community healing.
- TAIBU CHC introduced Kemetic yoga, an African-based movement system designed from African dance and ancestral teachings from the Kemetic people of ancient Egypt.^7^ This therapeutic practice was added to their social prescribing programs for Black seniors to provide an Afrocentric option rooted in the mind-body-spirit connection, holistic values and historical experiences of people of African descent. The program manager noted: “This not only nurtures physical health but also fosters a sense of cultural connection and self-awareness. Through social prescribing we have been able to offer our seniors a sense of belonging, enhancing their overall quality of life.”^8^

## Working collectively—thinking as a village

Meetings were organized to discuss the evaluation framework and to support implementing the projects. LogicalOutcomes, an evaluation consultancy with a background in community development, also hosted meetings. These discussions centred around Afrocentric principles and the limitations of the Westernized view in capturing spirituality as a determinant of health. Rather than making decisions as individuals, members of the group “think as a village,” consulting and collaborating with their colleagues.

Several tools have emerged, including the Wheel of Life,^9^ a tailored BFSP version of the client referral form from the pilot that includes eight life domains: spiritual, family, health, finance, career, friends, growth, and social life, plus “other” (if a category needs to be replaced). This tool aids in identifying and prioritizing relevant client concerns, as well as creating meaningful and affirming social prescriptions. Moreover, Black patients, who have often faced harm in the health care system, find additional benefits in working with Black health care staff with shared lived experiences.

## Evaluation framework

The evaluation framework is rooted in the Afrocentric principles and designed to ensure that client voices and experiences are brought into the centre of the evaluation, data collection, analysis and reporting. The framework, adapted from Hood et al.,^10^ is structured using the following criteria:

(1) History—place, people, program and evaluation’s role; traditions, cultural heritage;

(2) Location—evaluation recognizes intersections (individual, organizational, system levels and cultural context);

(3) Power—understanding privilege, attention to equity, social justice, disparities;

(4) Voice—addresses amplified and silenced voices; maps inclusion, exclusion;

(5) Connection—emphasizes relationships, time, place, universe; considers trust, accountability, responsibility; 

(6) Time—design emphasizes rhythm, pace, scheduling (before, during and after evaluation activities);

(7) Return—activities, findings that benefit the community;

(8) Flexibility—openness to change, new information, cultural perspectives; applies to evaluation design, process and products; and

(9) Reflection—apply evaluation principles, including self-scrutiny.

## Theory of change

A Theory of Change was developed through an extensive consultative process that included representatives from each CHC, Alliance staff and the consulting team. Working from the short-, medium- and long-term outcomes, the Theory of Change (“the Theory”) captures the activities, inputs and outputs that lead to better health outcomes for Black patients (available upon request).

Over the medium term (1–2 years), the aim is to enhance patient well-being by actions such as strengthening connections to community and traditional knowledge, reducing loneliness and fostering increased self-efficacy; these aims are achieved by improving mental health, trust and spiritual well-being, and by removing barriers to participation. An additional objective is to elevate the role of link workers by building a sense of belonging and ultimately decreasing turnover. The Theory also envisions improved health and well-being for the community and positive reports of health and well-being from clinicians. 

In the long term (3–5 years), the Theory anticipates improvements in patient health outcomes, as well as a deepened understanding of Afrocentric service provision. It envisions the embedding of Black-focused social prescribing into the practice at the four participating community health centres. 

## Challenges

Committing to an Afrocentric approach, BFSP brings about unique challenges that must be considered in order to build a shared sense of purpose and direction. With extensive participatory processes and consensus-building, significant time and effort were needed to ensure the project was consistent with shared principles. The following challenges were experienced during the early implementation:

(1) slow decision-making processes;

(2) challenges reaching an agreement regarding evaluation tools and processes;

(3) challenges in achieving trust-building and confidence among all key stakeholders;

(4) understanding local contexts and needs; and

(5) revising project approaches, documents and plans.

## Conclusion

To develop and deliver effective BFSP, it is crucial to consider carefully the context in which activities take place. This involves dedicating ample time for consultation and consensus-building, and a willingness to adapt to meet the community’s specific needs. The consultation and consensus-building process facilitates building evaluation tools rooted in Afrocentric principles, and considers individual and community perspectives.

The success of BFSP hinges on practitioners who deeply understand patient needs, interests, backgrounds and aspirations for improved health. By gaining insights into the Black community’s values and beliefs, we can foster trust, promote engagement and maximize overall effectiveness. Clients then feel a greater sense of belonging and are more likely to follow through with the social prescription referral. 

In conclusion, an inclusive and culturally sensitive approach to BFSP is vital for its success. By deeply understanding the community’s needs and incorporating Afrocentric principles, we can establish a meaningful connection with individuals, promote well-being and positively impact health outcomes.

## Acknowledgements

We are grateful to the Balsam Foundation for funding this work.

## Conflicts of interest

The authors declare they have no conflicts of interest.

## Authors’ contributions and statement

SR—conceptualization.

NP, NB—project administration.

JR—supervision. 

All authors contributed to analysis and writing.

The content and views expressed in this article are those of the authors and do not necessarily reflect those of the Government of Canada.
